# Amylose-Based Cationic Star Polymers for siRNA Delivery

**DOI:** 10.1155/2015/962941

**Published:** 2015-10-11

**Authors:** Tomoki Nishimura, Kaori Umezaki, Sada-atsu Mukai, Shin-ichi Sawada, Kazunari Akiyoshi

**Affiliations:** ^1^Department of Polymer Chemistry, Graduate School of Engineering, Kyoto University, Katsura, Nishikyo, Kyoto 615-8150, Japan; ^2^ERATO Akiyoshi Bio-Nanotransporter Project, JST, Katsura, Nishikyo, Kyoto 615-8150, Japan

## Abstract

A new siRNA delivery system using a cationic glyco-star polymer is described. Spermine-modified 8-arm amylose star polymer (with a degree of polymerization of approximately 60 per arm) was synthesized by chemoenzymatic methods. The cationic star polymer effectively bound to siRNA and formed spherical complexes with an average hydrodynamic diameter of 230 nm. The cationic 8-arm star polymer complexes showed superior cellular uptake characteristics and higher gene silencing effects than a cationic 1-arm polymer. These results suggest that amylose-based star polymers are a promising nanoplatform for glycobiomaterials.

## 1. Introduction

Since the discovery of RNA interference (RNAi) [1] and the achievement of gene silencing by synthetic small interfering RNAs (siRNAs) [2], siRNA has become established as a new tool for silencing target genes. siRNAs have, therefore, been widely recognized as novel potential therapeutics. To date, there has been considerable effort to develop siRNA therapeutics for treating viral infections and cancers [3]. For siRNA therapeutic applications, appropriate gene carriers are required because naked siRNA is readily degraded by nucleases. Moreover, siRNAs are too large and hydrophilic to cross cell membranes without a delivery method [4, 5]. To successfully deliver siRNAs, the carriers must penetrate biological barriers. Therefore, the development of gene carriers to efficiently deliver siRNAs remains an important challenge.

Various types of carriers for nucleic acids and other macromolecules have been developed, including viruses, nanoparticles, lipids, and polymers [6–9]. Though viral carriers are undeniably the most efficient for gene delivery, their use is encumbered by potential safety issues such as pathogenicity and immunogenicity. This has prompted development of nonviral carriers using biocompatible materials. Among the most commonly used polymer building blocks for these carriers are poly(ethylene glycol) [10, 11], poly(peptoid)s [12–14], and poly(amino acid)s [15].

Among materials under development for use as gene carriers, polysaccharides are one of the most promising because of low toxicity, biocompatibility, and biodegradability. Examples include amylopectin [16], chitosan [17], dextran [18], cellulose [19], pullulan [20], and schizophyllan [21]. We have described several series of spermine-modified cycloamylose derivatives that effectively delivered pDNA, siRNA, and CpG DNA* in vitro* and* in vivo* [22–24].

Amylose, a linear *α*(1,4) glucan with low polydispersity, can be enzymatically synthesized. We previously reported that a series of amylose-based star polymers can be prepared chemoenzymatically [25]. Spermine-modified 8-armed amylose star polymer, through its multivalent interactions with DNA, effectively catalyzed DNA strand exchange reactions. This multivalent character is also an important feature for a siRNA carrier. siRNA has a lower molecular weight than pDNA and strong interactions between the carrier and the siRNA would be required to form stable and compact complex nanoparticles. We therefore decided to take advantage of these properties and investigate amylose-based star polymers as potential siRNA carriers.

In this study, we report that a spermine-modified amylose-based star polymer acts as a siRNA carrier. siRNA-polymer complexes were characterized with respect to their sizes and charge ratios. In addition, their cytotoxicity and cellular uptake were evaluated by WST-8 assay and confocal laser-scanning microscopy (CLSM), respectively. Finally, delivery of a vascular endothelial growth factor specific siRNA (denoted by siVEGF) was evaluated at the mRNA level. For comparison, a monoarm glycopolymer with the same degrees of polymerization (D.P.) as the amylose arm of the star polymers was also characterized and evaluated for siRNA transfection efficiency.

## 2. Materials and Methods

### 2.1. Synthesis of Cationic Glyco-Star Polymers

Glycopolymers with a degree of polymerization of about 60 per arm were synthesized as described previously [25]. Spermine-modified glycopolymers were prepared by a conventional 1,1′-carbonyldiimidazole method. Briefly, carbonyldiimidazole (0.025 g) in DMSO (15 mL) was added dropwise to a solution of 8-arm glycopolymer (C8A, 0.10 g) in 10 mL dry DMSO at room temperature under argon and the reaction mixture was stirred for 5 h at room temperature. Spermine (0.32 g) in DMSO (10 mL) was then added to the reaction mixture and the mixture was stirred for 18 h at room temperature. The reaction solution was dialyzed against distilled water in a dialysis membrane (1000 MWCO) for 3 days and lyophilized to yield the solid products. One-arm cationic glycopolymer (C1A) was synthesized in an analogous manner.

### 2.2. siRNA and siRNA/Cationic Polymer Complexes

The siRNA species used were siRNA targeting murine VEGF (5′-CAG CUU GAG UUA AAC GAA CGU ACU U-3′, 5′-AAG UAC GUU CGU UUA ACU CAA GCU G-3′), denoted by siVEGF; nonsense siRNA (MISSION siRNA Universal Negative Control, Sigma-Aldrich, St. Louis, MO, USA), denoted by siCont; and Alexa488-labeled negative control siRNA (Invitrogen, Thermo Fisher Scientific, Grand Island, NY, USA).

To form siRNA/cationic polymer complexes, each siRNA (0.30 nM) and each cationic polymer (0.13 nM) was mixed gently and incubated for 30 min at room temperature.

### 2.3. Size and Zeta Potentials

Dynamic light scattering (DLS) and zeta potential (*ζ*) measurements were performed using a Malvern Zetasizer nano ZPS (Malvern Instruments Inc., USA) and data analyzed using Malvern software.

### 2.4. Transmittance Electron Microscopy (TEM)

Morphology of siRNA/cationic polymers complexes was observed with a TEM (HT-7700, Hitachi, Japan) at an acceleration voltage of 100 kV and a beam current of 20 *μ*A. Complexes were stained prior to TEM with 1 wt% phosphotungstic acid.

### 2.5. Confocal Laser-Scanning Microscopy (CSLM)

Renca cells were cultured in glass bottom culture dishes at a density of 1 × 10^5^ cells per dish at 37°C in 5% CO_2_/95% humidified air. After 24 h incubation, Alexa488-labeled siRNA/cationic polymer complexes were added. After 24 h, cells were observed with LSM 780 confocal fluorescence microscope (Carl Zeiss, Jena, Germany).

### 2.6. Cytotoxicity Assay

Renca cells were seeded at a density of 1 × 10^4^ cells/well on 96-well plates for 24 h in RPMI1640 medium supplemented with 100 U/mL penicillin, 100 *μ*g/mL streptomycin, and 10% FBS in advance. The culture media were replaced with fresh medium, and the polymers or siRNA/the polymer complexes were applied. After 24-hour incubation, the cell cytotoxicity was evaluated with Cell Counting Kit-8 (Dojin, Japan) according to the manufacturer's instructions. The absorbance was measured using a microplate reader with a filter of 450 nm. The cell viability was determined as a percentage of the absorbance of nontreated cells. The results were expressed as mean and standard deviation obtained from three samples.

### 2.7. siRNA Transfection* In Vitro* and RNA Isolation

Renca cells, cultured in RPMI1640 medium supplemented with 100 U/mL penicillin, 100 *μ*g/mL streptomycin, and 10% FBS, were seeded into 12-well tissue culture plates (1 × 10^5^ cells per well) at 37°C in 5% CO_2_/95% humidified air. After 24 h, siRNA/cationic polymer complexes, at concentrations as indicated in the figures, were added to the cells and incubation was continued under standard culture conditions. After 24 hours, total RNA was collected by RNeasy Micro Kit (Qiagen) according to the manufacturer's instructions.

### 2.8. Quantitative Real-Time PCR

For measurement of VEGF RNA expression, q-PCR was performed using LightCycler 480 Probe master (Roche). For the detection of VEGF mRNA, cDNA was synthesized from 500 ng of total RNA using the reverse reaction kit (ReverTra Ace qPCR RT Master Mix (Toyobo, Japan)) with the manufacturer's instruction. A LightCycler 480 Real-Time PCR System (Roche) was used for quantitative mRNA detection. The relative expression levels of mRNA were normalized to the expression of 18S ribosomal RNA. The expression of the gene was quantified by measuring cycle threshold (Ct) values and normalized using 2^−ΔΔCt^ Ct method relative to 18S ribosomal RNA.

## 3. Results and Discussion

**Figure 1:**
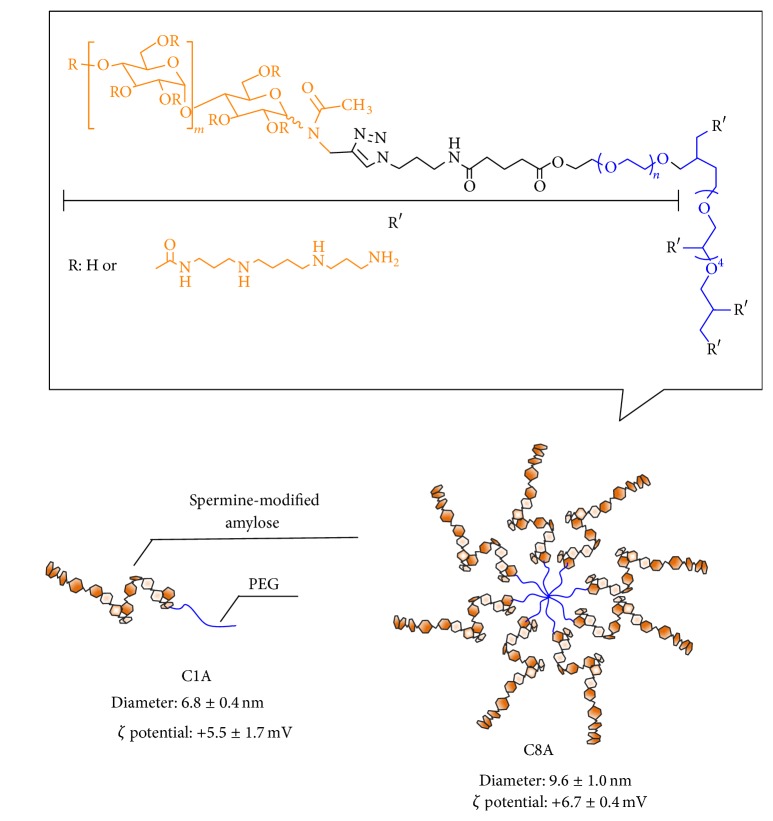
Chemical structures and illustrations of spermine-modified glycopolymers.

Glycopolymers were prepared by a chemoenzymatic method as reported previously [25]. Cationic spermine groups were introduced to the glycopolymers by a carbonyldiimidazole-mediated amide coupling reaction between the primary alcohol groups of amylose and the amino groups of spermine. The degree of substitution was about 30 spermine residues per 100 glucose units of the polysaccharide. The spermine functionalized mono- and octa-armed glycopolymers are denoted by C1A and C8A, respectively (Figure 1).

Polymer solutions in phosphate buffered saline (PBS, pH 7.4) were characterized with DLS and zeta potential analysis. The hydrodynamic diameters of C1A and C8A in PBS were 7 and 10 nm, respectively. The *ζ* potentials of C1A and C8A were +6 mV and +7 mV, respectively.

**Figure 2:**
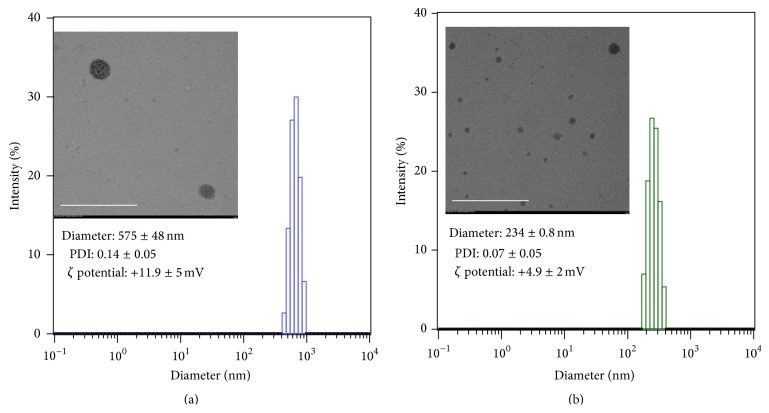
Size distributions of siRNA/C1A (a) and siRNA/C8A (b) complexes at C/P = 1.3 in PBS buffer. Inset: TEM images of the complexes. [polymer] = 0.098 *μ*g/*μ*L; [siRNA] = 0.032 *μ*g/*μ*L. The bars in the TEM images represent 1.0 *μ*m.

siRNA/C1A and siRNA/C8A complexes were prepared as described in Materials and Methods by mixing siRNA in nuclease-free H_2_O with the appropriate volumes of C1A or C8A solution (1.0 mg/mL), such that C/P ratios (ratio of cationic group in the glycopolymer to phosphate group in DNA) were 1.3. The size distributions and *ζ* potentials of the resulting complexes were determined (Figure 2). Both complexes showed a positive *ζ* potential (5–12) at C/P = 1.3. The siRNA/C8A complexes had an average hydrodynamic diameter of 234 ± 0.8 nm. In contrast, the siRNA/C1A complexes had an average hydrodynamic diameter of 575 ± 48 nm. By TEM observation, both complexes were spherical objects with size distributions comparable to what was found by DLS analysis. Compared with C1A, C8A had highly localized positive charges. This characteristic enables C8A to bind strongly to RNA. In fact, the binding affinity of C8A to DNA (20 base pairs) is 44 times greater than that of C1A [25]. The higher affinity of C8A for nucleic acids is believed to explain its formation of smaller complexes, as compared with those formed with C1A.

**Figure 3:**
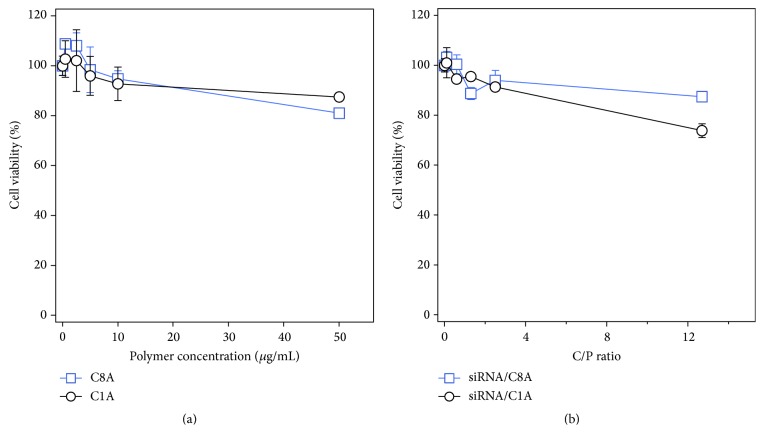
Cytotoxicity of the cationic polymers and siRNA/polymer complexes ([siRNA] = 0.1 nM). The complexes were incubated with Renca cells for 24 hours, and cell viability was evaluated by the WST-8 assay.

Since* in vitro* cytotoxicity of gene carriers is considered an important factor of biocompatibility, we investigated that cytotoxicity of our polymers with concentrations varied from 0 to 50 *μ*g/mL by WST-8 assay. As shown in Figure 3, exposure of the cells to the cationic polymers led to a slight decrease in cell viability (greater than 80%) for all polymer concentrations tested. Moreover, siRNA/cationic polymer complexes with various C/P ratios also showed no significant toxicity.

**Figure 4:**
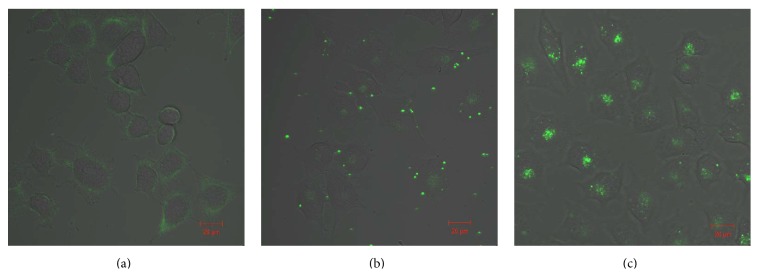
CLSM images of the glycopolymer delivery of Alexa488-labeled siRNA to Renca cells. (a) Naked siRNA, (b) siRNA/C1A complex, and (c) siRNA/C8A complex. Both complexes ([C1A] = 0.34 nM; [C8A] = 0.042 nM; [siRNA] = 0.1 nM, C/P = 1.3) were incubated with cells for 24 h. The bars represent 20 *μ*m.

Cellular uptake of siRNA and siRNA/cationic polymer complexes was then investigated in Renca cells with confocal laser-scanning fluorescence microscopy (CLSM). C1A and C8A solutions were mixed with Alexa488-labeled siRNA at a C/P of 1.3 and incubated for 30 min at room temperature. The resulting solutions were added to Renca cells and incubated in culture medium, as described in Materials and Methods, for 24 h. Cellular distributions of Alexa488-labeled siRNA, visualized by confocal laser-scanning microscopy (CLSM), are shown in Figure 4. Free siRNA was not internalized, remaining localized outside the cell. In contrast, green fluorescence was detected in the Renca cells using siRNA/cationic polymer complexes. Moreover, complexes formed with the C8A polymer resulted in greater cellular siRNA uptake than those with the C1A polymer.

**Figure 5:**
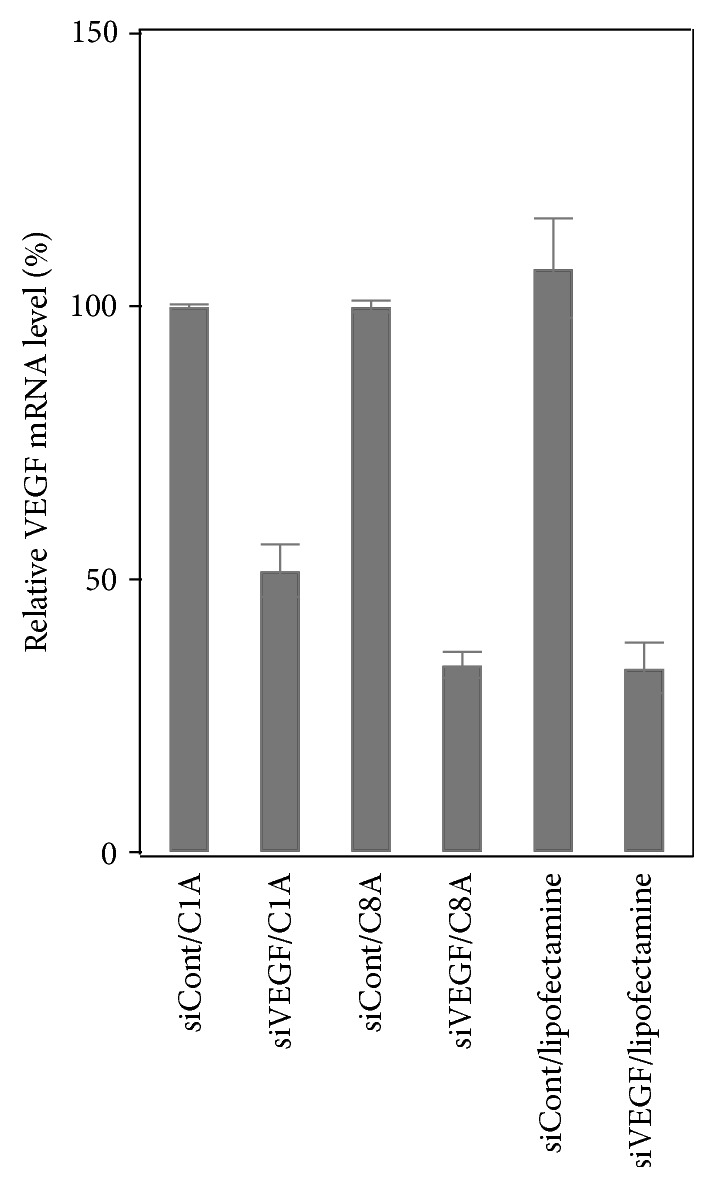
Gene silencing effect of nonsense siRNA (siCont)/C1A, sense siRNA (siVEGF)/C1A, siConc/C8A, siVEGF/C8A, SiCont/lipofectamine 2000, and siVEGF/lipofectamine 2000 complexes. [The polymer] = 5 *μ*g; [lipofectamine 2000] = 2 *μ*g. Dose of siRNA for the cationic polymers was 0.3 nM, while dose of siRNA for lipofectamine 2000 was 0.1 nM. C/P ratio was 1.3 for the siRNA/the cationic polymer complexes.

Next, we investigated RNAi effectiveness of the C1A- and C8A-based delivery systems. VEGF mRNA levels were evaluated by real-time RT-PCR analysis. Renca cells were treated with siRNA/C1A, siRNA/C8A complexes (C/P = 1.3, [siRNA] = 0.3 nM), and siRNA/lipofectamine 2000 as a positive control. As shown in Figure 5, VEGF mRNA levels were decreased after incubation with siVEGF/C8A or siVEGF/C1A complexes, relative to those in cells incubated with the corresponding nonsense siRNA complexes. Therefore, it is clear that both C1A- and C8A-based systems deliver siRNA into cells, enabling silencing of the target gene. The gene silencing effect of the C8A-based system (34% of control mRNA levels) was higher than that of C1A-based system (52% of control mRNA levels) and was almost comparable to that of lipofectamine 2000 system. Physical properties, including size, charge, and shape, of such complexes contribute greatly to cellular uptake efficiency [26, 27]. For the moment, the reason for the difference of the gene silencing efficiency is not clear. The relative sizes of the complexes might be attributed to this difference. The formation of smaller complexes between siRNA and the C8A polymer, as compared with the C1A polymer, might enhance cell internalization, leading to higher gene silencing efficiency.

## 4. Conclusions

In summary, we have demonstrated the utility of cationic glyco-star polymers as carriers for siRNA delivery. As compared with C1A, C8A can form more compact complexes with siRNA. The siRNA/C8A complexes were effectively internalized by cells and suppressed VEGF mRNA levels by about 65%. Our results show that the cationic glyco-star polymer is a promising platform for siRNA delivery.
